# Effectiveness of a Brief Mindfulness-Based Intervention of “STOP touching your face” During the COVID-19 Pandemic: a Randomized Controlled Trial

**DOI:** 10.1007/s12671-022-02019-x

**Published:** 2022-11-09

**Authors:** Jinsong Tang, Ling Wang, Tao Luo, Shiyou Wu, Zhenzhen Wu, Jianhua Chen, Chen Pan, Yunfei Wang, Yueheng Liu, Qinghua Luo, Xin Guo, Liqin Xie, Jun Zhou, Yunkai Sun, Wei Chen, Yanhui Liao

**Affiliations:** 1 Department of Psychiatry, Sir Run Run Shaw Hospital, Zhejiang University School of Medicine, 3 East Qingchun Road, Hangzhou, Zhejiang 310016 People’s Republic of China; 2 Florence Nightingale Faculty of Nursing, King’s College London, Midwifery & Palliative Care, London, UK; 3 Department of Psychology, the First Affiliated Hospital of Nanchang University, Nanchang, China; 4 Department of Social Medicine and Health Management, Xiangya School of Public Health, Central South University, Changsha, China; 5 The Third Affiliated Hospital of Guizhou Medical University, Duyun, Qiannan China; 6 Department of Psychiatry, the Second Xiangya Hospital, Central South University, Changsha, China; 7 National Clinical Research Center on Mental Disorders, Changsha, China; 8 Shanghai Clinical Research Center for Mental Health, Shanghai Key Laboratory of Psychotic Disorders, Shanghai Mental Health Center, Shanghai Jiao Tong University School of Medicine, Shanghai, China; 9 Department of Clinical Psychology, The Third Xiangya Hospital, Central South University, Changsha, China; 10 The First Affiliated Hospital of Chongqing Medical University, Chongqing, China; 11 Psychiatry Department, Renmin Hospital of Wuhan University, Wuhan, China; 12 Changsha Social Work College, Changsha, China; 13 Key Laboratory of Medical Neurobiology of Zhejiang Province, Hangzhou, China; 14 Addictions Department, Institute of Psychiatry, Psychology and Neuroscience, King’s College London, London, UK

**Keywords:** Face-touching behavior, Brief mindfulness-based intervention, Behavioral intervention, Transmission reduction, Infectious disease prevention, Brief online intervention

## Abstract

**Objectives:**

Avoiding touching the eyes, nose, and mouth (T-zone) is a strategy to reduce the spread of COVID-19. This study evaluated the effectiveness of a brief mindfulness-based intervention (MBI) named “STOP (Stop, Take a Breath, Observe, Proceed) touching your face” for reducing face-touching behavior.

**Methods:**

In this online-based, two-arm, wait-list, randomized controlled trial, eligible participants were randomly assigned to the intervention (*n* = 545) or control group (*n* = 545). The results of 60-min self-monitoring of face-touching behavior were reported before and after the intervention. Reduction of the percentage of T-zone touching was the primary outcome, and reduction of face-touching frequency was a key secondary outcome. Outcomes were analyzed on an intention-to-treat (ITT) basis with a complete case analysis (CCA).

**Results:**

ITT analysis revealed that the percentage of T-zone touching was significantly reduced by 8.1% in the intervention group (from 81.1 to 73.0%, RR = 0.901, OR = 0.631, RD =  − 0.081, *p* = 0.002), and insignificantly reduced by 0.6% in the control group (from 80.0 to 79.4%, *p* = 0.821). Fewer participants performed T-zone touching in the intervention group than in the control group (73.0% vs. 79.4%, RR = 0.919, OR = 0.700, RD =  − 0.064, *p* = 0.015) after the intervention, and there was a greater reduction of T-zone touching frequency in the intervention group than in the control group [mean ± SD: 1.7 ± 5.13 vs. 0.7 ± 3.98, mean difference (95% CI): 1.03 (0.48 to 1.58), *p* < 0.001, Cohen’s *d* =  − 0.218]. The above results were further confirmed by CCA.

**Conclusions:**

This brief mindfulness-based intervention was potentially effective at reducing the spread of COVID-19 and could be further investigated as an intervention for preventing other infectious diseases spread by hand-to-face touching.

**Trial Registration:**

ClinicalTrials.gov NCT04330352.

**Supplementary Information:**

The online version contains supplementary material available at 10.1007/s12671-022-02019-x.

The coronavirus disease 2019 (COVID-19) pandemic, caused by the SARS-CoV-2 virus, is a severe public health emergency. According to the World Health Organization (WHO) prevention guidelines for COVID-19 and a systematic review and meta-analysis (Chu et al., 2020), three simple and common interventions to reduce COVID-19 spread are maintaining physical distance from others, wearing face masks, and avoiding touching the eyes, nose, and mouth (T-zone). Face-touching behavior is thought to increase the risk of COVID-19 infection via the transfer of droplets from the hands to the T-zone. However, face touching is a common behavior. The average face-touching frequency ranges from approximately 16 (Nicas & Best, 2008) to 23 (Kwok et al., 2015) times per hour, and 42.2% (4) (Morita et al., 2019) to 44% (3) (Kwok et al., 2015) of face touching involves the T-zone. Even clinicians touch their T-zone frequently, ranging from 0 to 105 times (a mean of 19 times) within 2 h (Elder et al., 2014). A systematic review including 10 single-arm observational studies found that the average frequency of self-touching of the T-zone was 68 times per hour (Rahman et al., 2020). The frequency and duration of self-touching behavior have also been associated with cognitive and emotional demands (Grunwald et al., 2014; Mueller et al., 2019), and linked to information processing and production (Harrigan, 1985).

Face-touching behavior, as an important part of our nonverbal communication, is often done automatically with little or no self-awareness (Harrigan et al., 1986). It is a particularly challenging behavior to control, but this can be achieved by targeting an alternative behavior. Raising self-awareness of habituated face-touching behavior may help individuals avoid touching their faces with contaminated hands, and thereby prevent the spread of infection.

Based on empirical evidence, a search for studies of mindfulness-based intervention (MBI) to reduce face-touching behavior conducted from 1980 to June 2021 was performed using the following search terms: “mindfulness-based intervention” OR “behavior intervention” AND “face-touching behavior” OR “face touching” OR “hand-to-head behavior.” This search focused on seven databases (China National Knowledge Infrastructure, Wanfang Data Resource, Google Scholar, EMBASE, Cochrane, Medline, and PsycInfo). No studies involving evaluation of the effectiveness of MBI or behavioral intervention for reducing face-touching behavior were identified. However, a cross-sectional study reported that mask-wearing reduced face-touching behavior (Chen et al., 2020) and a review indicated the effectiveness of habit reversal training (HRT) for reducing risky behaviors like face touching (Heinicke et al., 2020). However, there is a need for high-quality clinical trials to demonstrate the effectiveness of HRT for reducing face-touching behavior.

The present study was designed as a brief mindfulness-based intervention (MBI) named “STOP (Stop, Take a Breath, Observe, Proceed) touching your face” based on a mindfulness practice developed by Kabat-Zinn (2003) and used by Smalley and Winston (2010) to reduce the risk of infection via contaminated hands. We hypothesized that the use of this MBI would achieve a greater reduction of face touching than a wait-list control intervention.

## Methods

### Participants

Participants were recruited via online social media, including WeChat and QQ. Research assistants screened potential participants who contacted the research team. Participants were eligible for inclusion if they were aged 18 years or older and had access to online services. We excluded participants who were unable to read and write Chinese and had already received training on “not touching your face” (all of whom were healthcare providers). Informed consent was obtained electronically from each participant before baseline data collection.

The sample size assessment was mainly based on the results of RCTs of different types of online brief MBI for behavioral changes, such as reducing alcohol consumption (Kamboj et al., 2017), quitting smoking or reducing nicotine craving (Garrison et al., 2020), or positive psychological changes, such as improving wellbeing (Howells et al., 2016) and reducing symptoms of stress, anxiety, and depression (Cavanagh et al., 2013). The current study estimated that the reduction in the percentage of T-zone touching would range from 5 to 10% in the brief intervention group and 2 to 4% in the control group. A total of 562 participants would provide 80% confidence in detecting the above difference in the percentage of T-zone touching between the study groups, with 5% significance. Considering that online interventions often have a high dropout rate, ranging from 20% (Buntrock et al., 2016) to 30% (Cavanagh et al., 2013), this study had a final target sample size of over 1000 participants.

### Procedure

After reporting baseline information and the first 60-min self-monitored face-touching behavior (by the same link), a researcher individually randomized and assigned participants to the intervention or wait-list control condition. Randomization was performed via https://CRAN.R-project.org/package=randomizeR. No stratification variables were chosen in the randomization. The investigator who provided intervention was not blinded to the group. After receiving the intervention, the second 60-min self-monitoring of face-touching behavior was reported by different links to detect group allocation. The investigators who analyzed the data were blinded to the allocations until they completed all analyses. All participants from the control group received the mindfulness-based intervention after completion of the trial.

The current study included three procedures: (1) pre-intervention: complete the first 60-min self-monitoring of face-touching behavior and baseline information; (2) intervention: receive a brief mindfulness-based STOP touching your face intervention or control messages; and (3) post-intervention: complete the second 60-min self-monitoring of face-touching behavior. The repeat measurement of face-touching behavior was performed at least 1 h after the first self-monitoring. Details of the procedures have been described previously (Liao et al., 2020).

#### Data Collection

The baseline and post-intervention data collection took place from April 2, 2020, to July 2, 2020. Eligible participants were invited to complete a battery of questions at baseline (baseline information and record of the first 60-min self-monitoring of face-touching behavior) and post-intervention (record of the second 60-min self-monitoring of face-touching behavior) through Wen Juan Xing (Sojump, Shanghai, China, www.sojump.com), a professional online data collection platform that can be used for online RCTs (Fitzpatrick et al., 2018). All data were automatically collected through the internet. One user-specified Excel file of a pre-intervention face-touching behavior report with baseline information and two separate files for two groups of post-intervention face-touching behavior reports were downloaded from the database. Data were monitored by the data monitoring committee of the hospital. All personal data were anonymized.

#### Interventions

The four-component behavioral intervention was developed by mindfulness-based cognitive and behavior theory Kabat-Zinn, 2003; Smalley & Winston, 2010). The intervention aimed to increase self-awareness and/or concentration associated with face-touching behavior, to help individuals avoid touching their faces with contaminated hands to prevent the spread of infection. The “STOP” practice is one of the most popular mindfulness-based practices (Black et al., 2015; Kabat-Zinn, 2003; Smalley & Winston, 2010). It is a short, simple, and informal mindfulness practice that allows us to take a pause to check in on how we are doing or feeling. The STOP practice can assist in shifting from an “autopilot” and distracted state to one of awareness and concentration (Baer & Krietemeyer, 2006).

The practice of STOP touching your face cultivates self-awareness and the capacity to pause before responding to spontaneous behavior, such as touching one’s own face (Vago & Silbersweig, 2012). The brief behavioral intervention of STOP touching your face in the current study was developed by Dr. Y. Liao (for full text in Chinese and English, see Supplementary file 1; a 5-min audio description is published in the protocol as Data supplement 1) (Liao et al., 2020). There are four successive steps in this brief intervention: S = stop, stop whatever you are doing or whatever you are going to do (e.g., mouth touching, nose pinching, or eye rubbing), remind yourself to stop for a minute; T = take, take a deep breath to connect yourself with your body; O = observe, observe what is happening to you at this moment (e.g., feel distracted or anxious, feel itchy or tingling on any part of your face), and P = proceed, proceed with whatever you were doing before or you are doing now (e.g., proceed with or stop eye rubbing). For a more detailed description of the STOP touching your face program, see the report by Liao et al. (2020). All participants were encouraged to practice the STOP touching your face regularly after the end of the trial.

Participants from the intervention group received the mindfulness-based STOP touching your face program remotely (mainly via WeChat and QQ). All participants from the intervention group received the text and audio of the program. They were encouraged to practice the skill until they confidently and naturally performed it, and were required to initially practice for at least 15 min after learning it. Participants could contact the researchers (by phone or via social media) if they had any questions related to the study, despite the lack of face-to-face interaction between the researchers and the participants during the study period.

Members of the comparison group received message to thank them for their participation, encourage them to complete the study, and remind them of the STOP touching your face program after the end of this study. All participants from the control group received the STOP touching your face program remotely (via WeChat) after the trial.

### Measures

One of the three simple interventions promoted by the WHO to reduce the spread of COVID-19 is not just avoiding touching the face, but reducing the frequency of touching the T-zone. In other words, to prevent the spread of COVID-19, avoiding touching the T-zone would be more effective than reducing face-touching frequency in general. Thus, the primary outcome of this study was set as the reduction of the percentage of participants who touched their T-zone (eyes, nose, and mouth). The secondary outcomes were reductions of face-touching frequency, including the whole face (eyes, nose, mouth, ears, cheeks, chin, neck, forehead, and hair), T-zone (eyes, nose, and mouth), and non-T-zone (ears, cheeks, chin, neck, forehead, and hair); the factors (demographic characteristics, psychological traits of mindfulness) associated with face-touching frequency; and the factors associated with reduction of face-touching frequency. The percentage of T-zone touching was calculated as the number of participants who touched any of the eyes, nose, and mouth during a 60-min session of self-monitoring of face-touching relative to the total number of participants in the intervention group or the control group. The percentage of non-T-zone touching was calculated as the number of participants who touched any of the ears, cheeks, chin, neck, forehead, and hair during a 60-min session of self-monitoring of face-touching relative to the total number of participants in the intervention group or the control group. The percentage of overall face touching was calculated as the number of participants who touched any of the eyes, nose, mouth, ears, cheeks, chin, neck, forehead, and hair during a 60-min session of self-monitoring of face-touching relative to the total number of participants in the intervention group or the control group. The face-touching frequency was calculated as the total number of face touches (including the eyes, nose, mouth, ears, cheeks, chin, neck, forehead, and hair) during a 60-min period. Reduction of the percentage of T-zone touching was calculated as the percentage of participants who touched their T-zone during the first 60-min period (pre-intervention) minus the percentage of participants who touched their T-zone during the second 60-min period (post-intervention). Reduction of face-touching frequency was calculated as the total number of face touches during the first 60-min period (pre-intervention) minus the total number of face touches during the second 60-min period (post-intervention).

All participants were required to report self-observation or self-monitoring results of face-touching behavior using a standardized scoring sheet to tally the frequency of hand-to-face contacts, and which area of the face was touched. The Five Facet Mindfulness Questionnaire (FFMQ) (Baer et al., 2006; de Bruin et al., 2012; Deng et al., 2011) was used to measure the general tendency to be mindful in daily life, using five related facets (observing, describing, acting with awareness, non-judging internal experience, and non-reactivity to internal experience). The Edinburgh Handedness Inventory (EHI) (Oldfield, 1971; Yang et al., 2018) was used to measure handedness.

### Data Analyses

Intention-to-treat (ITT) analysis (McCoy, 2017) was applied in this study. Thus, all randomized participants were included in the statistical analysis and analyzed according to the initially assigned group. There were no interim analyses. Data were analyzed using R software (R) and IBM SPSS Statistics (SPSS). Descriptive statistics were applied for baseline information; a two-sample *t* test or Mann–Whitney *U* test (for continuous variables) and *χ*^2^ test (for categorical variables) were applied to compare baseline information, reduction of the percentage of T-zone touching, and reduction of face-touching frequency between the study groups. Analysis of covariance (ANCOVA) was applied with control for baseline variables. In the ANCOVA model, the dependent variable was the reduction of face-touching behavior. The pre-intervention measure of the total number of face touches was controlled as a covariate and intervention was a fixed factor. This model assessed the group differences of reduction of face-touching frequency after the intervention after accounting for pre-intervention values. Pearson’s correlation or regression analysis (linear and binary regression model) was used to explore any factor that was associated with face-touching behavior at baseline and reduction of face-touching behavior in the intervention group and in the control group. The last observation carried forward (LOCF) method, a commonly used statistical approach to handle repeated measurement data if some follow-up data are missing (Lachin, 2016), was applied to handle incomplete or missing data (assuming that there were no changes of face-touching behavior from pre-intervention to post-intervention). In addition, a complete case analysis (CCA) was performed in which participants with missing information at the follow-up were excluded. A threshold of two-sided *P* < 0.05 was applied to determine statistical significance.

## Results

**Fig. 1:**
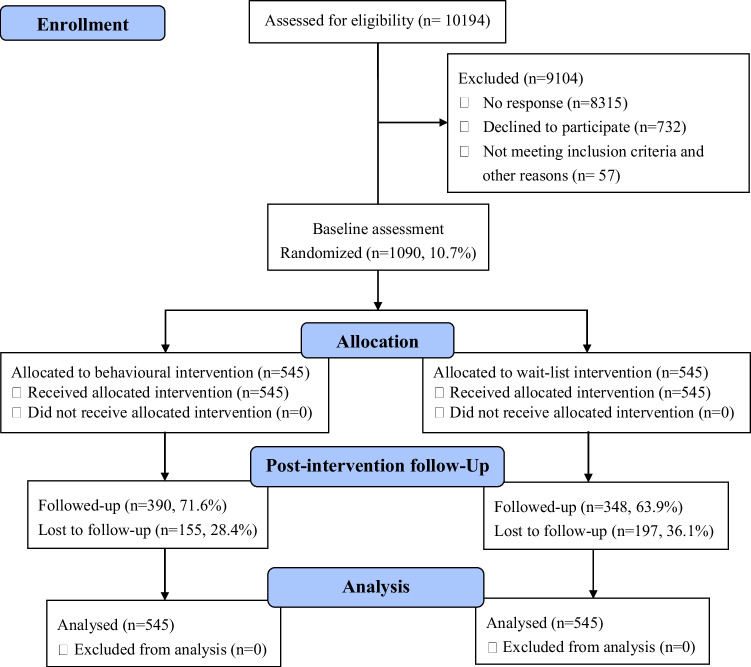
Flow diagram for STOP touching your face study

Figure 1 shows the study flow. During the recruitment period of the trial, from April 2, 2020, to July 2, 2020, 10,194 participants were referred to the trial, of whom 1090 (10.7%) participants were assigned to the “STOP touching your face” intervention group (*n* = 545) or to the wait-list control group (comparison, *n* = 545) after reporting the first 60-min session of self-monitoring of face-touching behavior (pre-intervention) and providing complete baseline information. Among them, 71.6% (*n* = 390) of participants from the intervention group and 63.9% (*n* = 348) from the control group reported the second 60-min session of self-monitoring of face-touching behavior (post-intervention). A total of 1090 participants were included in the final analysis. We asked all participants to report safety concerns if any arose. No safety concerns were identified in this study.

### Baseline Information

Demographic information, elements of mindfulness, and face-touching behavior at baseline in the STOP touching your face intervention group and the wait-list control group are shown in Table 1. The two groups were matched for FFMQ and EHI at baseline. There were also no differences between the study groups in T-zone touching frequency and the percentage of T-zone touching. Compared with the control group, the intervention group was 2 years younger on average, was less commonly in a nonmedical occupation, had a lower rate of being married, tended more to live in rural areas, and had higher overall face and non-T-zone touching frequencies at baseline.

**Table 1:** Baseline information between the study groups

	Control group (*n* = 545)	Intervention group (*n* = 545)
Age^*^, mean ± SD	30.8 ± 10.19	28.7 ± 9.18
Male, %	32.8	29.7
Education (years), M ± SD	16.7 ± 3.07	16.5 ± 3.05
Nonmedical^*^, %	42.8	23.9
Married^*^, %	46.1	35.6
Rural region^*^, %	9.4	13.8
Never-smoker, ex-smoker, %	90.3, 2.8	90.3, 2.0
EHI, mean ± SD	80.2 ± 36.80	77.4 ± 41.75
Right hander^#^, %	87.0	85.3
Meditation experience, %	18.0	17.8
Meditation (month), mean ± SD	2.1 ± 8.41	2.1 ± 10.48
FFMQ total score	48.0 ± 4.87	47.6 ± 5.40
Face-touching frequency at baseline, mean ± SD		
Overall face^*^	15.3 ± 16.25	18.7 ± 20.26
T-zone	4.5 ± 5.56	5.4 ± 7.68
Non-T-zone^*^	10.8 ± 12.06	13.2 ± 14.90
Reported T-zone touching at baseline, %	80	81.1

Face-touching frequency: the total times of face touching (including the eyes, nose, mouth, ears, cheeks, chin, neck, forehead, and hair) during a 60-min period before intervention; Overall face: including the eyes, nose, mouth, ears, cheeks, chin, neck, forehead, and hair; *T-zone* the mucus membranes of the eyes, nose, and mouth, *Non-T-zone* the ears, cheeks, chin, neck, forehead, and hair

*FFMQ* The five-facet mindfulness questionnaire

^*^p < 0.05

^#^The Edinburgh Handedness Inventory (EHI) was used to assess handedness

### T-Zone Touching by Intervention

**Fig. 2:**
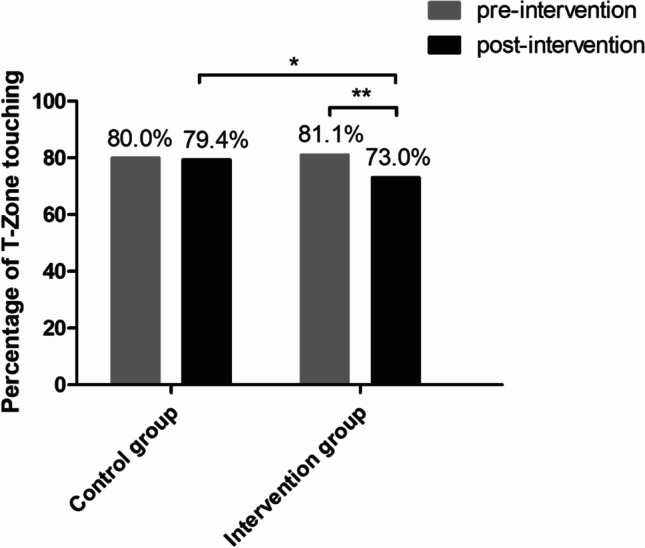
The percentage of T-zone-touching participants between pre- and post-intervention in the study groups. *p < 0.05; **p < 0.01

Figure 2 shows the percentages of T-zone touching in the study groups before and after the intervention. First, a reduction in the percentage of T-zone touching over time was found in each study group. Based on ITT and LOCF (assuming that there was no change of T-zone touching from pre-intervention to post-intervention for missing data), the percentage of T-zone touching was significantly reduced by 8.1% in the intervention group [from 81.1% pre-intervention to 73.0% post-intervention, risk ratio (RR) = 0.901, odds ratio (OR) = 0.631, risk difference (RD) =  − 0.081, *p* = 0.002], and insignificantly reduced by 0.6% in the control group (from 80.0% pre-intervention to 79.4% post-intervention, *p* = 0.821). Then, the percentage of T-zone touching was also compared between the study groups after the intervention. ITT analysis showed that fewer participants undertook T-zone touching in the intervention group than in the control group (73.0% vs. 79.4%, RR = 0.919, OR = 0.700, RD =  − 0.064, *p* = 0.015).

CCA (upon excluding missing data) showed similar results. The percentage of T-zone touching was significantly reduced by 10.3% in the intervention group (from 81.1% pre-intervention to 70.8% post-intervention, RR = 0.873, OR = 0.564, RD =  − 0.103, *p* < 0.001) and insignificantly reduced by 1.6% in the control group (from 80.0 to 78.4%, *p* = 0.576). In addition, fewer participants undertook T-zone touching in the intervention group than in the control group (70.8% vs. 78.4%, RR = 0.902, OR = 0.665, RD =  − 0.077, *p* = 0.018).

### Face-Touching Frequency

The effectiveness of the STOP touching your face intervention was assessed by comparing the group differences in the reduction of face-touching frequency between the intervention and control groups. We first compared the mean reduction of face-touching frequency after intervention between the study groups. Upon assessments by ITT and LOCF (assuming no reduction of face-touching frequency from pre-intervention to post-intervention for missing data), reductions of face-touching frequency in the overall face, T-zone, and non-T-zone were identified in the two groups, as shown in Table 2. By CCA (with missing data excluded), this study also showed significant differences between the two groups (see Table 3).

**Table 2:** Reduction of face-touching frequency between the “STOP touching your face” intervention group and the control group by ITT analysis

	Intervention group (*n* = 545), mean ± SD	Control group (*n* = 545), mean ± SD	Mean difference (95% CI)	*p* value*	Cohen’s *d*
T-zone	1.7 ± 5.13	0.7 ± 3.98	1.03 (0.48 to 1.58)	<0.001	− 0.218
Non-T-zone	4.1 ± 9.13	1.6 ± 6.67	2.49 (1.54 to 3.44)	<0.001	− 0.313
Overall face	5.7 ± 12.14	2.2 ± 9.49	3.52 (2.23 to 4.82)	<0.001	− 0.321

*ITT* intention to treat, assuming no reduction of face-touching frequency from pre-intervention to post-intervention for missing data

^*^p < 0.05

**Table 3:** Reduction of face-touching frequency between the “STOP touching your face” intervention group and the control group by CCA

	Intervention group (*n* = 390), mean ± SD	Control group (*n* = 348), mean ± SD	Mean difference (95% CI)	*p* value*	Cohen’s *d*
T-zone	2.4 ± 5.93	1.1 ± 4.94	1.33 (0.54 to 2.11)	0.001	− 0.237
Non-T-zone	5.6 ± 10.36	2.4 ± 8.23	3.22 (1.87 to 4.56)	<0.001	− 0.34
Overall face	8.0 ± 13.70	3.5 ± 11.70	4.55 (2.71 to 6.38)	<0.001	− 0.352

*CCA* complete case analysis: missing data were excluded

^*^p < 0.05

Considering that some baseline information did not match between the two groups, this study further explored the effectiveness of the brief MBI of the STOP touching your face program for reducing face-touching frequency by ANCOVA. Based on ITT or CCA, this study found significant effects of group [intervention group and control group] (*F* = 13.85, *p* < 0.001, *η*^2^*p* = 0.236, or *F* = 9.56, *p* = 0.002, *η*^2^*p* = 0.228) on the reduction of face-touching frequency. The current study also found that there were no significant differences in participants’ characteristics (age, occupation, and marital status, all *p* > 0.1), except for living in an urban or rural area (*F* = 7.079, *p* = 0.008, *η*^2^*p* = 0.168, or *F* = 12.14, *p* = 0.001, *η*^2^*p* = 0.257) and overall face-touching frequency at baseline (*F* = 492.38, *p* < 0.001, *η*^2^*p* = 1.405, or *F* = 616.85, *p* < 0.001, *η*^2^*p* = 1.834), in the reduction of face-touching frequency.

### Factors Associated with Face-Touching Behavior

The current study explored the association of age and mindfulness with the face-touching frequency by linear regression analysis and found that younger participants are more likely to increase face-touching frequency (see Table 4).

**Table 4:** MLR model predicting increase in face-touching frequency at baseline

Variable	*B*	CI	*p* value*
Age (years old)	− 1.215	− 1.862 to − .0568	0.000
Years of education	− .102	− 0.343 to 0.138	0.404
Length of meditation (months)	− .006	− 0.819 to 0.807	0.988
FFMQ total score	− .072	− 0.393 to 0.248	0.657

Multiple linear regression (MLR) model

*CI* 95% confidence interval, *FFMQ* The five-facet mindfulness questionnaire

^*^p < 0.05

This study found that approximately 80% of participants touched their T-zone at baseline assessment. Therefore, we explored whether any demographic factors (sex: male vs. female, age: 18–29 vs. ≥ 30, education: undergraduate degree or lower vs. graduate degree or higher, occupation: medical vs. nonmedical profession, marital status: married vs. unmarried, residence: urban vs. rural, smoking status: current smoker vs. never or former smoker, meditation experience: with vs. without, handedness: left or mix vs. right) were associated with T-zone face-touching behavior. Binary logistic regression analysis reveals that only age was a risk factor for T-zone face touching. Compared with older individuals (≥ 30 years old), young adults (18–29 years old) had twice the risk of exhibiting T-zone touching behavior [OR = 2.029, 95% confidence interval (CI) = 1.145 to 3.597, *p* = 0.015].

### Factors Associated with Reduction of Face-Touching Frequency

Pearson’s correlation analyses revealed no associations of demographic or mindfulness-related characteristics (including age, residence, smoking status, meditation experience, and psychological traits of mindfulness) with reduction of face-touching frequency in the control group. However, there was a significant association between reduction of face-touching frequency and age in the intervention group (*r* =  − 0.24, *p* < 0.001 with ITT analysis, or *r* =  − 0.25, *p* < 0.001 with CCA), but other characteristics and length of STOP practice were not associated with reduction of face-touching frequency. Regression analysis further revealed that reduction of face-touching frequency was only associated with age (*t* =  − 4.870, 95% CI =  − 0.536 to − 0.228, *p* < 0.001) in the intervention group. Then, we further compared the reduction of face-touching frequency between those aged ≥ 30 (*n* = 272, 69.7%) and aged < 30 (*n* = 118, 30.3%) in the intervention group, and found that younger participants exhibited greater reductions than their older counterparts [mean ± SD: 9.9 ± 14.24 vs. 3.6 ± 11.25, mean difference (95% CI) = 6.270 (3.615 to 8.924), *p* < 0.001, Cohen’s *d* =  − 0.47].

## Discussion

We evaluated the effectiveness of the STOP touching your face program for reducing face-touching behavior on the basis of mindfulness and cognitive behavioral principles using a large sample of participants from the general Chinese population. This study demonstrated that a brief and simple intervention in the form of the STOP touching your face program significantly reduced T-zone touching behavior, a type of behavior that can increase the risk of COVID-19 and other infectious diseases.

The current study showed that approximately 80% of participants reported T-zone touching, and they touched their T-zone four to five times during a 60-min session of self-monitoring of face-touching at baseline. These results indicate that face-touching is a frequent, habituated behavior, which is consistent with findings in other studies (Kwok et al., 2015; Nicas & Best, 2008). The current study found significant differences in the percentage of T-zone touching between pre- and post-intervention in the STOP touching your face intervention group (decreased by 8.1% based on ITT, and 10.3% on CCA), but not in the control group. There were also group differences in the reduction of face-touching frequency between the intervention group and the control group. The frequencies of T-zone, non-T-zone, and overall face touching in the intervention group all showed reductions that were twice those in the control group. Our findings also strongly support the notion that young people have a higher likelihood of performing T-zone touching behavior, but they may gain a larger benefit from the STOP touching your face program than their older counterparts.

A strength of this study is its use of an RCT with a large sample size to evaluate the effectiveness of a theoretical framework-guided (mindfulness-based cognitive behavior theory) brief intervention of STOP touching your face during the COVID-19 outbreak. The findings in the current study are similar to those in ITT analyses and CCA. Because CCA can underestimate the potential bias caused by missing data from loss to follow-up, and an ITT principle may prevent this potential bias, the consistency of these results is a significant strength of this study. The robustness of the findings is also promoted by the support of secondary outcomes relating to the difference in reduction of face-touching behavior between the study groups.

As a theoretical framework-guided RCT with a large sample size evaluating the effectiveness of the STOP touching your face program during the COVID-19 outbreak, this study demonstrated that this free, brief, and simple mindfulness-based behavior-change intervention significantly reduced T-zone touching behavior. At the policy level, our findings are important for updating guidelines for preventing COVID-19 and other infectious diseases. At the general population level, in addition to vaccinations and pharmacotherapeutics, there is a high demand for measures to prevent the spread of COVID-19 by changing human behavior, particularly via easy-to-understand behavioral strategies like the STOP touching your face program that can be delivered online.

### Limitations and Future Research

Nevertheless, when interpreting these findings, the limitations of the current study should be taken into account. First, the current study was conducted during the COVID-19 pandemic, so we applied a WeChat-based intervention with self-monitoring of face-touching behavior by participants, rather than recording by researchers. However, self-monitoring has been recommended to promote physical activity (Aittasalo et al., 2006), improve mental health and wellbeing (Bakker & Rickard, 2018), and enhance medication management and patient outcomes (Lancaster et al., 2018). Thus, self-monitoring of face-touching behavior itself may increase participants’ awareness of habituated face-touching behavior, which may lead to an increase or decrease of this behavior during the 60-min observation period. From this perspective, this limitation has important implications: it makes the results less reliable because we do not know exactly what the observed decrease means. Furthermore, a systematic review revealed over- or underreporting of physical activity by self-reported measures compared with the findings obtained by direct methods (Prince et al., 2008). The self-reported measures used in this study may thus have over- or underestimated the actual face-touching behavior. However, our study found very similar results during the first and second 60-min observations of face-touching behavior in the control group.

As another limitation, as in other similar online MBI studies (Cavanagh et al., 2018), the percentage of participants lost to follow-up was relatively high in both groups, especially in the control group. To compensate for this high dropout rate, we applied an ITT principle to prevent potential bias caused by missing data from loss to follow-up, and completer analyses were also reported. A third limitation is that the participants may have gained fewer benefits from the short-term STOP touching your face practice provided online than from face-to-face and long-term MBI. However, several RCTs have shown that brief online MBI can improve mindfulness, decrease stress, alleviate symptoms of anxiety and depression (Cavanagh et al., 2013; Zhang et al., 2021), and reduce levels of paranoia in a non-clinical population (Shore et al., 2018). The results from the current study also indicate the effectiveness of the STOP touching your face intervention provided in an online format. A final limitation is that this study did not measure how wearing a mask would change participants’ face-touching behavior. One study found that wearing a mask may reduce face‐touching behavior among healthcare professionals (Lucas et al., 2020).

## Funding Sources

This study was funded by Zhejiang University Special Scientific Research Fund for COVID-19 Prevention and Control (2020XGZX046), the “Hundred Talents Program” funding from Zhejiang University, and a K.C. Wong Postdoctoral Fellowship to study at King’s College London (YHL). The funders of the study had no role in study design, data collection, data analysis, data interpretation, or writing of the report.

## Supplementary Information

Below is the link to the electronic supplementary material.

Supplementary file1 (DOCX 12 KB)

## Data Availability

All data are available in the Supplementary Materials.
