# Supplementary material for: Effectiveness of a Brief Mindfulness-Based Intervention of “STOP touching your face” During the COVID-19 Pandemic: a Randomized Controlled Trial
Source: Mindfulness (N Y). 2022 Nov 9;13(12):3123–33. doi: 10.1007/s12671-022-02019-x (PMC9645303; doi:10.1007/s12671-022-02019-x)
Supplement: Supplementary file 1 — Supplementary file1 (DOCX 12 KB) [file 12671_2022_2019_MOESM1_ESM.docx]

**Supplementary file 1**

“STOP (Stop, Take a Breath, Observe, Proceed) touching your face” program in Chinese and English
